# Sputtered thin film deposited laser induced graphene based novel micro-supercapacitor device for energy storage application

**DOI:** 10.1038/s41598-024-62192-y

**Published:** 2024-07-15

**Authors:** Sourav Sain, Suman Chowdhury, Sayantan Maity, Gurupada Maity, Susanta Sinha Roy

**Affiliations:** 1 Department of Physics, School of Natural Sciences, Shiv Nadar Institution of Eminence (SNIoE), Deemed to be University, Delhi-NCR, Greater Noida, 201314 India; 2 Department of Physics and Astrophysics, University of Delhi, Delhi, 110007 India; 3 Department of Chemistry, School of Natural Sciences, Shiv Nadar Institution of Eminence (SNIoE), Deemed to be University, Delhi-NCR, Greater Noida, 201314 India

**Keywords:** Sputtering, Hafnium oxide (HfO_2_), Laser Induced Graphene (LIG), Micro supercapacitor devices (MSC), Flexible electrodes, Supercapacitors, Electrochemistry

## Abstract

Pioneering flexible micro-supercapacitors, designed for exceptional energy and power density, transcend conventional storage limitations. Interdigitated electrodes (IDEs) based on laser-induced graphene (LIG), augmented with metal-oxide modifiers, harness synergies with layered graphene to achieve superior capacitance. This study presents a novel one-step process for sputtered plasma deposition of HfO_2_, resulting in enhanced supercapacitance performance. Introducing LIG-HfO_2_ micro-supercapacitor (MSC) devices with varied oxygen flow rates further boosts supercapacitance performance by introducing oxygen functional groups. FESEM investigations demonstrate uniform coating of HfO_2_ on LIG fibers through sputtering. Specific capacitance measurements reveal 6.4 mF/cm^2^ at 5 mV/s and 4.5 mF/cm^2^ at a current density of 0.04 mA/cm^2^. The LIG-HfO_2_ devices exhibit outstanding supercapacitor performance, boasting at least a fourfold increase over pristine LIG. Moreover, stability testing indicates a high retention rate of 97% over 5000 cycles, ensuring practical real-time applications.

## Introduction

Micro-supercapacitors (MSCs) are a category of energy storage devices known for high power density and facilitating rapid charging-discharging processes. These are well-suited for devices that require quick bursts of energy. The utilization of an MSC is significant for its electrochemical performance as well as its flexibility for miniaturization to establish portable energy devices^1–3^. Supercapacitors can be classified into Electrical Double Layer Capacitor (EDLC) and Pseudocapacitor (PC). These two are characterized by the electrostatic interaction and definite redox reactions at the electrolyte–electrode interface, respectively. The consolidated advantage of both can be enjoyed in a hybrid supercapacitor (HSC) which takes in the longevity and recyclability from EDLC and the contribution of additional Faradaic current to the overall capacitance value^4–6^. In general, composite materials are used for the development of HSC^7,8^. It is highly desirable to retain the qualities of the individual components in HSC and the ductility for using the resultant material in flexible devices^1–3,7,8^.

Laser-induced graphene-based (LIG) devices represent a noteworthy subset of MSCs that effectively fulfill such requirements, offering seamless integration with microelectronic devices^9^. They have large surface areas and microporous structures to facilitate active charge accumulation and transport. The fabrication method of LIG includes one-step lithography-free laser irradiation of polymers containing aromatic carbon chains. This avoids high-temperature treatment and the use of toxic chemicals^10^. However, challenges persist, especially the need for the development of robust and scalable production methods. Additionally, optimizing specific capacitance while ensuring stability and reliability remains a problem since graphene-based materials suffer from low energy density^11^. On the other hand, metal oxides of transition metals have widely been used as the redox-active component^7,8^. They can substantially increase the Faradaic current and strengthen the ion reachability for being ionic in nature but possess a different drawback of high charge transfer resistance and sheet resistance. Disadvantages of both carbonaceous materials and metal oxide nanoparticles can be overcome through a proportionate blending following a careful synthetic protocol, and a successful HSC can be achieved^12^. The ductility of transition metals is an added advantage over s and p-block elements to make them more perfect for flexible devices.

The oxides and hydroxides of Ru have emerged with the best HSC performance so far among other transition metals. But it is environmentally hazardous and economically not sustainable for its extortionate price and instability^13^. Hence, oxides of Mn, Fe, Co, Sn, etc. have been used more frequently^14^. Transition metals having larger d-electrons can undergo variable oxidation states, resulting in multiple redox cycles during the electrochemical processes. This may bring down the performance for a long cycle. Extra care should be taken also if the metals are highly suspectable to air and moisture. In this regard, light transition metals of group III and group IV appear to be promising. Their highest oxidation states, + 3 and + 4 respectively, contain vacant d-orbitals which can be useful for extra charge accommodation without enduring any repulsion^15^. To date, oxides of Ti and Zr have been composited with graphene or graphitic materials to fabricate supercapacitor electrodes and they have come up with very good results^14,16^. The next metal in group IV, Hf, may be a costly element but its properties indicate it to be a potential candidate for HSC. These three metals have identical outer shell configurations (d2) but increasing atomic radius induces electron shell penetration effect from Ti to Zr to Hf^15,16^. Additionally, HfO_2_ is well known for its high dielectric constant, and therefore, has excelled already as an exquisite performer for nanoscale energy devices^17^. La, having one d-electron less than Hf and a member of group III, has also been utilized as HSC^18^. But Hf appears to be even more profitable over La for the presence of 14 underlying 4f.-electrons, due to which the lanthanide contraction effect makes the oxide of Hf more stable^15,17^.

The application of HfO_2_ as HSC, combined with any carbonaceous materials, is not very common in the literature. Hydrothermally prepared reduced graphene oxide (rGO) and HfO_2_ nanomaterials have exhibited enhanced supercapacitor performance, which was nearly double the performance compared to pure HfO_2_^19^. While rGO-based electrodes show promising results, their integration poses challenges and involves complex, multi-step chemical synthesis processes. These have been tried to be overcome by Co-doping in HfO_2_/rGO^20^. However, in the present article, we target to avoid the use of heavier transition metals, as explained earlier. Hydrothermal synthesis of graphene and HfO_2_ nanocomposite has been reported, but the material has not been tested for any application^21^. Its extensive characterization indicates that prevention of the dielectric loss and tangent loss during the synthesis is very tricky and it is well-known that HfO2 is not expected to work as an HSC without preventing those. All the studies lead us to find an alternative way. To the best of our knowledge, the HfO_2_/LIG composite has not been synthesized yet. An innovative approach revolves around LIG synthesis achieved by irradiating polyimide sheets on both sides and stacking them, showcasing a substantial enhancement in the specific capacitance of the end product^22^. On the other hand, ultrathin high-dielectric HfO_2_ was reported to be deposited on flexible polyimide substrates through sol–gel spin coating and the resultant flexible device displayed excellent capacitive behaviors^23^. Recently, sputtered thin-film-based MSC devices have been developed and applied successfully featuring FeWO_4_/MnO_2_-based electrodes^24^. Hence, the development of HfO_2_/LIG is supposed to pave away the limitations and complications of the hydrothermal method and in-situ fabrication techniques.

Earlier, our group reported the development of LIG along with its various applications. We have already explored the surface functional group modifications and metal oxide incorporation for sensing and supercapacitor applications^25,26^. In the present study, we utilized an innovative sputtered deposition system tailored for the development of flexible LIG, specifically designed for MSC applications. The LIG was meticulously crafted through a rapid laser engraving process, resulting in the formation of 3D porous graphene-based flexible electrodes. After this, HfO_2_ was precisely deposited via sputtering, with optimization of deposition parameters. The impact of varying oxygen content during deposition, relative to Argon, on supercapacitor performance was systematically explored. In 2019, DFT calculations indicated that the graphene band gap can be tuned in the presence of ferroelectric HfO_2_ and the role of oxygen is very important here^27^. The authors concluded the study that, thermal treatment on oxygen may be beneficial in controlling the band gap. Inspired by this, we emphasized the regulation of oxygen flow during the synthesis of HfO_2_/LIG. The influence of HfO2 deposition on graphene was probed using Density Functional Theory (DFT) through Vienna Ab-initio Simulation Package (VASP) simulations. To assess stability under demanding conditions, Galvanostatic Charge–Discharge (GCD) at a very high current density was employed. This multifaceted approach provides a comprehensive understanding of the fabrication process and performance characteristics, yielding valuable insights for the advancement of flexible MSC technology.

## Experimental details

### Materials and reagents

All the chemicals are laboratory reagent (LR) grade. We brought in 127-µm polyimide (PI) sheets from Cole-Parmer. Potassium hydroxide (KOH) and Sulfuric acid (H_2_SO_4_) are purchased from Fisher Scientific. Polyvinyl alcohol (PVA) was purchased from Sigma-Aldrich. All of the studies conducted in this article used de-ionized (DI) water with a resistance of ~ 18.2 MΩ.

### Fabrication and characterization

Supercapacitor performance was examined in an autolab potentiostat/galvanostat 302N equipment (MetrohmAutolab B.V. Utrecht, Netherlands) through the techniques like cyclic voltammetry (CV), galvanostatic charging-discharging (GCD), and electrochemical impedance spectroscopy (EIS). MV Laser, India, provided the CO_2_ laser cutting and engraving machine used to produce the LIG-based electrodes. The contact angles were determined through a contact angle measuring instrument by APEX, India. The surface morphology of LIG-HfO_2_ was scrutinized through a Field emission Scanning Electron Microscope (FESEM) from JEOL, USA. A micro-Raman spectrophotometer (STR) was used to measure the Raman spectrum for vibrational modes. X-ray photoelectron Spectroscopy (XPS) is measured for the four HfO_2_-LIG samples using Mg-Kα beam (1253.6 eV) radiated from a VersaProbe III (PHI, USA) system. The selected regions for Hf4f., C1s, and O1s are scanned repeatedly times to obtain smooth peaks. CasaXPS (version 2.3.22PR1.0) software is used for analyzing the XPS data.

The fabrication of LIG was carried out by laser irradiation of the PI sheet at a power output of ~ 5.5 W using a 90 W CO_2_ laser. Before laser irradiation, the PI sheet was properly rinsed and fixed on the stage. The interdigitated electrodes were designed using RDWorksV8 (License free) used to operate the laser system. The line spacing and scanning seed were set at 0.07 mm and 180 mm/s respectively. The interdigitated LIG was then transferred to the sputtering system. The HfO_2_ was deposited on LIG using an Ar flow rate of 40 SCCM, varying the deposition time for optimization. The RF power was set at 80 W for all deposition purposes. The selected optimal deposition time was then employed in conjunction with oxygen and Argon at different flow rate ratios. The Argon to oxygen flow rates were further adjusted, with ratios of 1:1, 2:1, and 4:1 on the LIG surfaces, resulting in LIG-HfO_2_ MSC devices. The final fabricated interdigitated electrodes were then coated with PVA-H_2_SO_4_ gel electrolyte giving rise to LIG-HfO_2_-based micro-supercapacitor (MSC) devices. The real image is also shown in Fig. S1 (Supporting Information).

### Electrochemical methods

To assess supercapacitive performance, the sputtered LIG-HfO_2_ interdigitated (ID) MSC devices were coated with a gel electrolyte comprising PVA-H_2_SO_4_. The gel electrolyte was prepared by dissolving 1 g of PVA in 10 mL of deionized (DI) water, with constant stirring at 85 °C. The solution was vigorously agitated for several hours until it transformed into a transparent gel. After cooling, H_2_SO_4_ was incrementally added drop by drop. For optimization of deposition time, cyclic voltammetry (CV) and galvanostatic charge–discharge (GCD) scans were conducted at a scan rate of 40 mV/s and 0.05 mA/cm^2^ within a potential range of 0 to 0.8 V. The following equations were used to calculate the capacitor properties of our devices.

1 $${C}_{CV}= \frac{{\int }_{{V}_{a}}^{{V}_{c}}i\left(V\right)dV}{2 \times A \times \vartheta \times \Delta V}$$

2 $${C}_{GCD}= \frac{I \times \Delta t}{A \times \Delta V}$$

3 $${E}_{A}= \frac{{C}_{GCD} \times {\left(\Delta V\right)}^{2}}{2 \times 3600}$$

4 $${P}_{A}= \frac{{E}_{A} \times 3600}{\Delta t}$$

5 $${\eta }_{C}= \frac{{t}_{D}}{{t}_{C}}$$

where ${C}_{CV}$ and ${C}_{GCD}$ is the specific capacitance calculated from the cyclic voltammetry and galvanostatic charge-discharge curves with ${\int }_{{V}_{a}}^{{V}_{c}}i\left(V\right)dV$ being the area under the curve, A is the area of the electrode surface, $\vartheta$ is the scan rate in mV/s, $\Delta t$ discharge time, $\Delta V$ is the potential window, ${E}_{A}$ energy density, and ${P}_{A}$ power density. ${\eta }_{C}$ is the coulombic efficiency with charging time ${t}_{C}$ and discharging time ${t}_{D}$ in seconds.

### Computational Details

In all the numerical calculations, we have made use of the ab-initio DFT code as provided by the Vienna Ab-initio Simulation Package (VASP)^28–31^. The ion–electron interaction was described using the projector-augmented wave (PAW) method^32^. All the simulations were performed employing generalized gradient approximation (GGA) with Perdew-Burke–Ernzerhof (PBE) exchange and correlation^33^. 520 eV mesh cutoff energy has been set in the expansion of plane wave basis sets. The Brillouin zone (BZ) has been sampled by a Γ-centered 7 × 7 × 1 k-points for self-consistent total energy computations and geometry optimizations. The electronic ground state energy convergence criteria have been set by 10^–5^ eV. The structure was geometrically relaxed until the unbalanced force components converged below 0.04 eV/Å. Perpendicular to the graphene sheet, more than 15 Å vacuum space was used to prevent artificial interactions between the periodic images.

## Results and discussion

### Surface morphology

**Figure 1:**
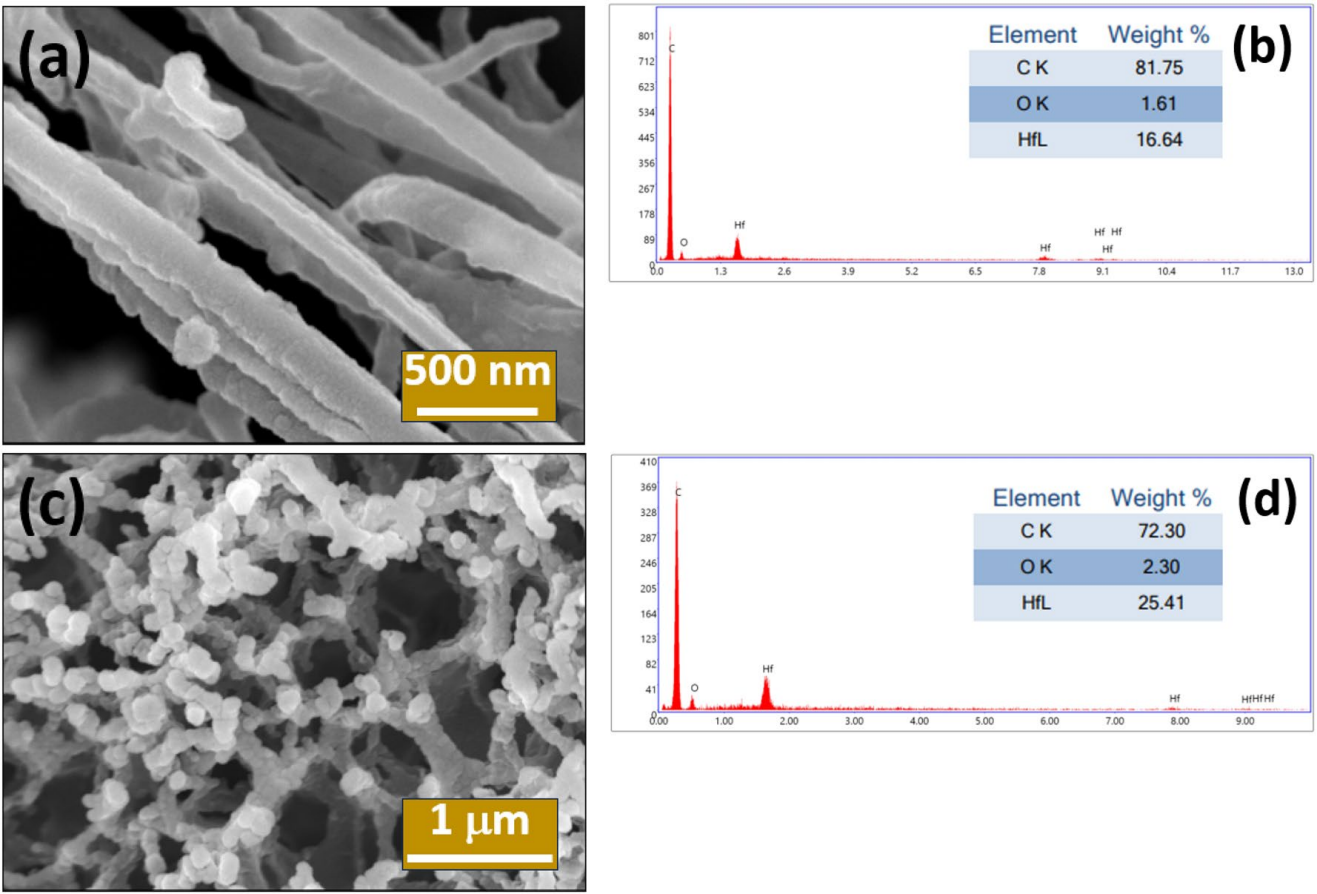
FESEM images of HfO_2_ deposited on LIG at (**a**) 1 h, and (**c**) 1.5 h, and elemental mapping of LIG-HfO_2_ shown in (**b**) 1 h, and (**d**) 1.5 h.

The LIG-HfO_2_ MSC devices were fabricated with a laser power of ~ 5 W in a continuous scanning mode as mentioned in previous section "Fabrication and characterization". When the PI sheet was rapidly irradiated with a laser it allowed the trapped gases to escape forming a 3D porous structure. Our earlier work (see Fig. 1b in reference^25^) features Fe-SEM images of pristine LIG. The LIG-HfO_2_-based electrodes were fabricated by the sputtering system in the presence of argon as mentioned in previous section "Fabrication and characterization". The deposition thickness was mainly governed by deposition time and was optimized along with the study of electrochemical performance as discussed later. Uniform deposition on the surface of LIG-HfO_2_ fibers was found to be up to 1 h of deposition time as shown in Fig. 1a. The EDS spectroscopy also confirms the presence of HfO_2_ on the surface of LIG fibers with an elemental percentage of 16.6% compared to carbon of 81.7% as shown in Fig. 1b. As the deposition time increased there was too much surface deposition causing agglomeration on LIG-HfO_2_ fibers as shown in Fig. 1c. This agglomeration may increase the dielectric loss and tangential loss of the deposited dielectric material HfO_2_ rendering its performance. The agglomeration was observed at a deposition time of 1.5 h. This decreases the supercapacitor performance as discussed later. The hafnium elemental concentration was 25.4% compared to carbon of 72.3% as shown in Fig. 1d. FESEM investigation confirms uniform deposition on the LIG fiber surface up to a deposition time of 1 h.

### Surface functional groups

**Figure 2:**
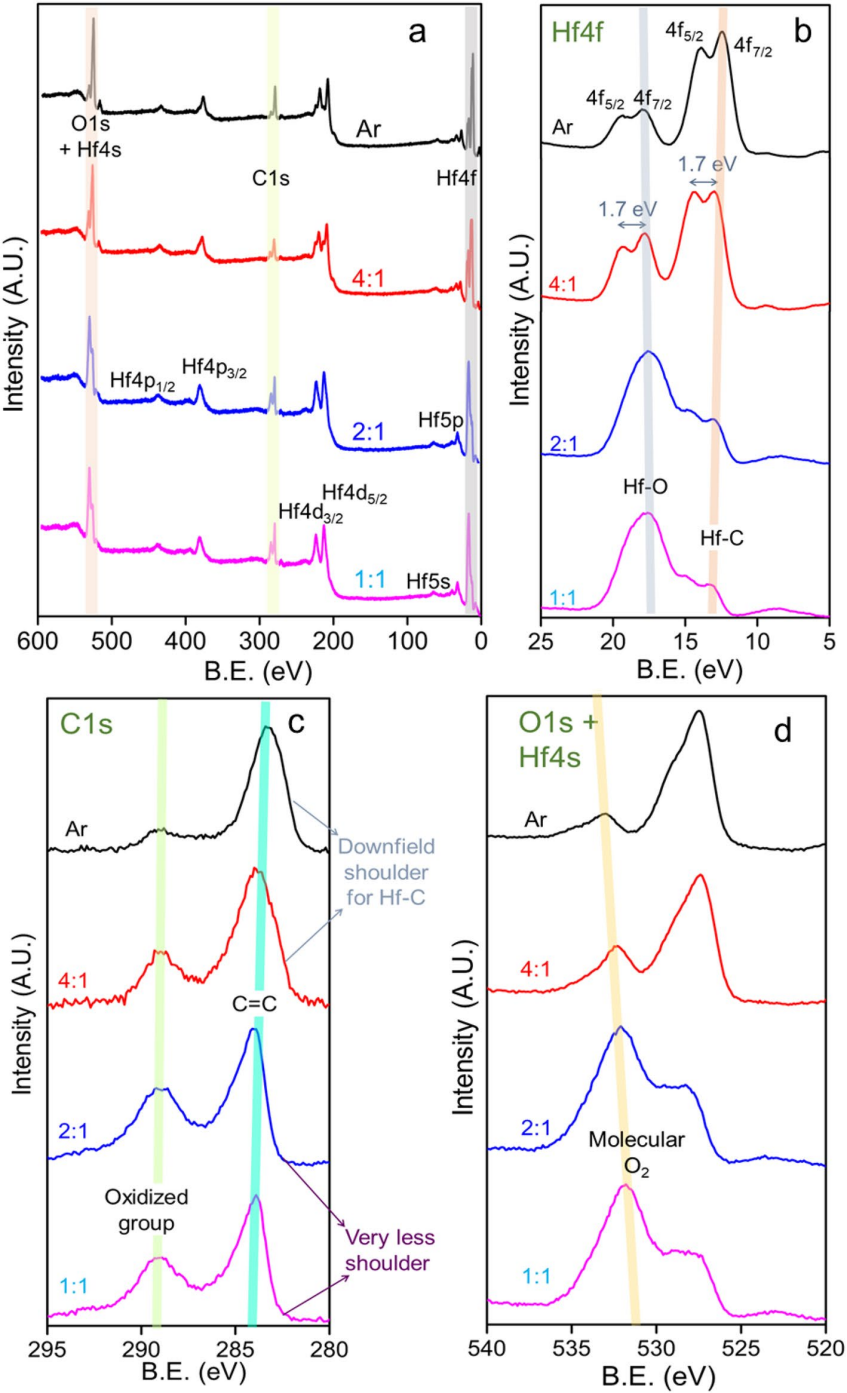
XPS spectroscopy of HfO_2_ deposited on LIG (**a**) Survey scan, (**b**) Hf 4f., (**c**) C 1s, and (**d**) O 1s.

XPS survey spectrum of the four samples reveals that the major constructing element is Hf (Fig. 2a). All the major and minor peaks from different energy levels of Hf are assigned according to previous reports^34,35^. A doublet between 290 and 284 eV is assigned for C_1S_ and the 4s peak of Hf merges with the O_1s_ XP line between 528–533 eV region. The upper surface distribution of HfO_2_, leaving the LIG layer underlying, causes the dominance of Hf peaks over C. Further, the core lines of the three elements are scrutinized more closely. The Hf_4f_ band is split between 4f_7/2_ and 4f_5/2_ levels of energy, 1.7 eV apart (Fig. 2b). The binding energy (BE) for the Hf–O bond is expected to reside between 16.7 and 18.1 eV, according to the report by N. Ohtsu et al.^34^. The appearance of the downfield doublet between 13 and 15 eV is due to the formation of the Hf-C bond as reported by Rodenbücher et al.^35^. Upon moving from Ar → 4:1 → 2:1 → 1:1, the Hf–O intensity increases and the doublet combines to one single broad peak in 2:1 and 1:1. The intensity of Hf-C decreases in the same sequence and the band remains as a shoulder in 2:1 and 1:1. This indicates excess oxidation of Hafnium during deposition on LIG in presence of more oxygen. This is further supported by the core level XP lines of C_1s_ (Fig. 2c). The main peak of C_1s_ is at 284.2 eV which is a characteristic peak for C=C of graphene. This is accompanied by a flat small peak at ~ 288–289.2 eV for the oxidized groups of carbon with double bonded oxygen (e.g., C=O, C–OOH) which is intensified under more oxygen exposure^36^. In Ar and 4:1, a prominent downfield shoulder is observed at ~ 282–282.5 eV, which is associated with the Hf-C bond^35^. In accordance with the Hf_4f._ peaks, the Hf-C band decreases in the rest of the two samples and is observed at the peak base only. Since the O_1s_ (Fig. 2d) peak is merged with the Hf_4s_ and there are both molecular O_2_ adsorbed on the HfO_2_ surface and bonded oxygen with Hf and C, it is not possible to distinguish the components separately^37^. But, the peak near ~ 532 eV BE is supposed to be for molecular oxygen which increases upon more oxygen exposure. A red shift of this peak is observed simultaneously. This is consistent with the previous report and may be due to the charging effect^37^.

### Electrochemical optimization for deposition of Hafnium oxide

**Figure 3:**
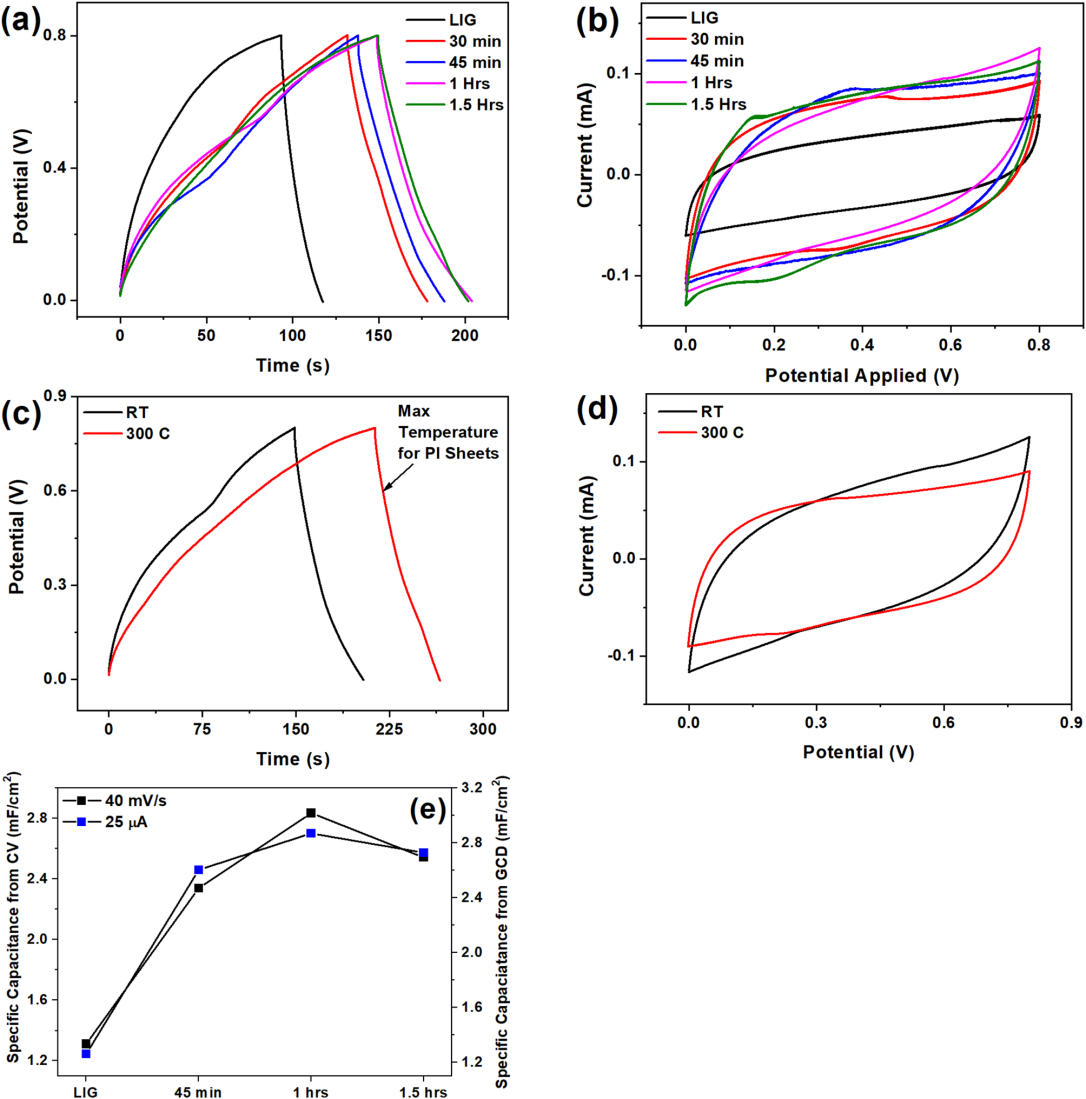
Optimization for sputtered deposition for hafnium oxide on LIG surface (**a**) GCD plots, (**b**) CV plots, Temperature dependant study for supercapacitor performance for LIG-HfO_2_ (**c**) GCD plots, (**d**) CV plots, (**e**) specific capacitance from CV and GCD plots corresponding to deposition time on LIG.

Interdigitated electrodes based on Laser-Induced Graphene (LIG) often demonstrate excellent electrochemical double-layer performance. In Fig. 3a, the capacitive performance of LIG-HfO_2_ electrodes is depicted at various current densities. The calculations of specific capacitance from CV and GCD graphs were performed using Eqs. (1) and (2). The cyclic voltammetry (CV) curves illustrate quasi-rectangular shapes for each HfO_2_ derivative electrode. The incorporation of dielectric layers, such as graphene oxide, further enhances supercapacitance properties^38^, with room temperature deposition of HfO_2_ exhibiting a notably higher dielectric constant of 18.2, confirming its suitability for supercapacitor-based devices^39^. Fig. 3b reveals the superior supercapacitor performance of HfO_2_ deposited LIG compared to pristine LIG, evidenced by increased areal capacitance in CV curves. The charge passed during the electrochemical process, represented by the area under the cyclic voltammetry (CV) scans, correlates directly with the integral of current over time. Larger areas in CV scans confirm enhanced supercapacitor properties, denoting increased charge storage capacity. The charge storage mechanism may be also due to the presence of Hafnium on the surface of LIG having some redox reaction given by^40.^

$$\begin{gathered} {\text{Hf}} + {\text{2H}}_{{2}} {\text{O}} \to {\text{Hf}} - \left( {{\text{OH}}} \right)_{{2}} + {\text{2H2e}}^{ - } \hfill \\ {\text{Hf}} - \left( {{\text{OH}}} \right)_{{2}} + {\text{2OH}}^{ - } \to {\text{2H}}_{{2}} {\text{O}} + {\text{Hf}} - {\text{O}}_{{2}} + {\text{2e}}^{ - } \hfill \\ {\text{Hf}} - {\text{O}}_{{2}} \to {\text{Hf}} + {\text{O}}_{{2}} \hfill \\ \end{gathered}$$

This mechanism may also be suppressed and not prominently visible due to the presence of a thin layer of HfO_2_ on the LIG surface. Earlier published work has also shown a diminishing effect of oxidation–reduction peak in CV curves due presence of a lesser fraction of Hf on the graphene surface^41^. The Galvanostatic Charge–Discharge (GCD) plots also demonstrate a longer discharge time for HfO_2_ deposited LIG compared to LIG alone, indicating superior micro-supercapacitor performance. The extended discharge duration implies greater energy storage capacity, making it suitable for applications requiring continuous power delivery. As deposition time increases, the thickness of the HfO_2_ layer improves overall device performance. However, supercapacitor performance reaches saturation after one hour of deposition, potentially due to increased electron transfer resistance. The specific capacitance at 1 h is 2.8 mF/cm^2^, whereas, at 1.5 h, it decreases to 2.5 mF/cm^2^ at 40 mV/s, indicating a saturation point in supercapacitor performance (Fig. 3b). Higher temperatures result in thinner deposition layers, as illustrated in Fig. 3d, leading to a decline in specific capacitance. Notably, the charge–discharge time is more asymmetric at 300 °C than at room temperature, suggesting that LIG-HfO_2_ exhibits superior performance at room temperature, simplifying electrode fabrication as shown in Fig. 3c. Figure 3e illustrates the optimization of deposition time for LIG-HfO_2_ based devices in hafnium oxide formation. The specific capacitance gradually rises with increased deposition time up to 1 h, followed by a decline due to active material overload. This result directly correlates with the FE-SEM images discussed in the earlier section "Surface morphology". The decrease in specific capacitance with an increase in deposition time may be attributed to the agglomeration increasing the dielectric loss and tangential loss of the deposited active material as observed in Fig. 1c. LIG-HfO_2_ based devices exhibit outstanding supercapacitance properties, reaching 2.8 mF/cm^2^ at a current density of 0.05 mA/cm^2^.

### Effect of oxygen plasma treatment and Coulombic efficiency

**Figure 4:**
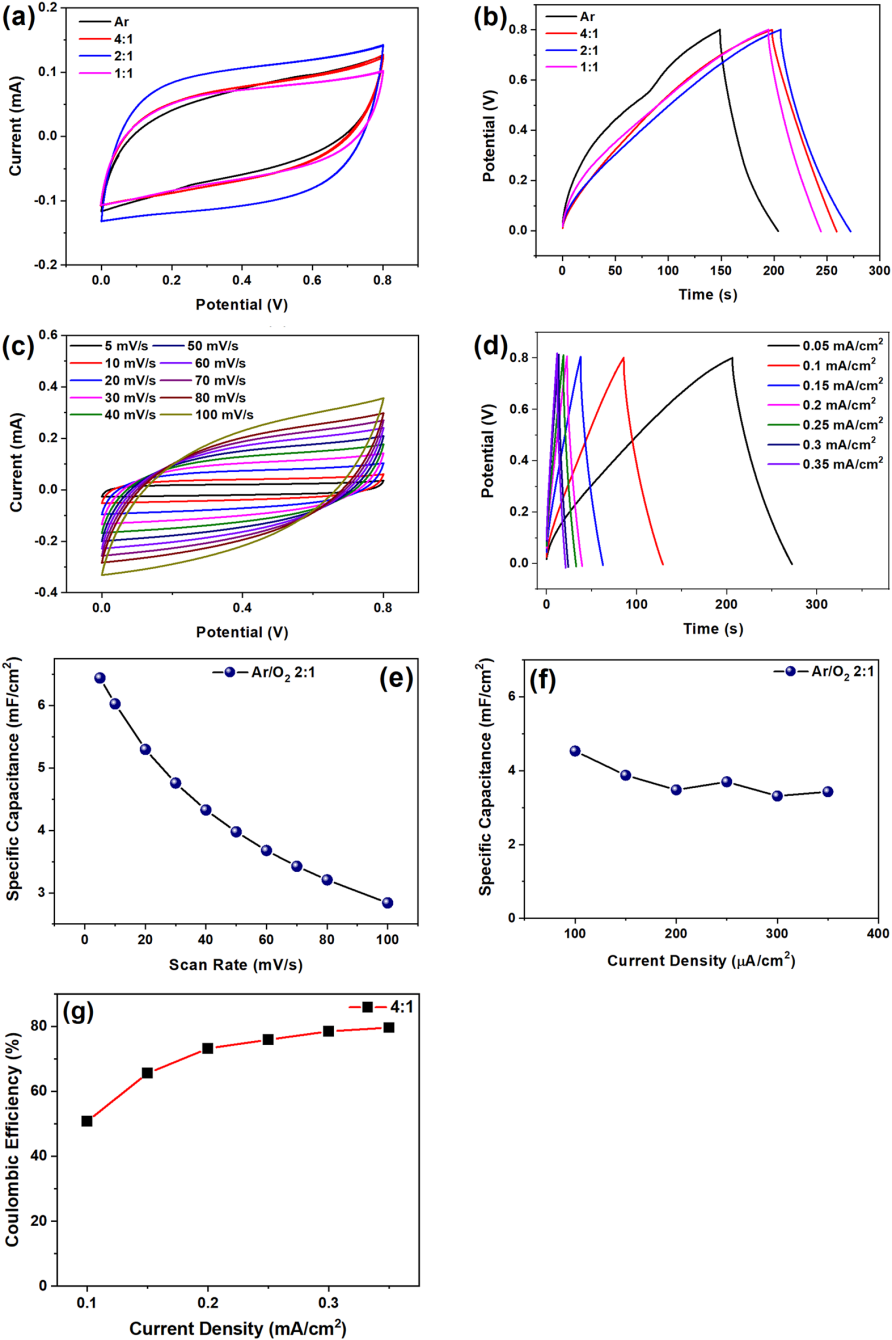
Optimization of oxygen functional groups with different argon by oxygen ratio for electrochemical performance of (**a**) CV plots, (**b**) GCD plots, (**c**) CV voltammograms with variation of scan rates for LIG-HfO_2_ corresponding to Ar:O_2_ 2:1, (**d**) GCD with variation of current density, (**e**) specific capacitance from CV plots with variation of scan rates, (**f**) specific capacitance with variation of current density, (**g**) Coulombic efficiency plot with current density at the Ar:O_2_ ratio 4:1.

The voltammetric current shows a direct proportionality to the scan rate, and as polarization increases, the cyclic voltammetry (CV) curve gradually deviates from rectangularity. At higher scanning speeds, H^+^ ions face increased difficulty penetrating the HfO_2_, resulting in polarization. Lower scanning rates allow H^+^ ions sufficient time to reach the HfO_2_ surface and be injected into it. The properties of the electrode surface and the graphene shape significantly influence the CV curve. The concentration and types of oxygen functional groups have a notable impact on the capacitive performance of graphene electrochemical supercapacitors. Generally, an increase in the concentration of oxygenated species corresponds to enhanced specific capacitance^42^. In Fig. 4a, various CV plots corresponding to different oxygen gas flow rates are illustrated. Oxygen was purged at increasing flow rates, in conjunction with argon, at argon-to-oxygen ratios of 4:1, 2:1, and 1:1. The area under the CV plot increases as the argon-to-oxygen flow rate transitions from 4:1 to 2:1, followed by a gradual decrease from 2:1 to 1:1. The maximum specific capacitance was observed at a ratio of 2:1, reaching 6.4 mF/cm^2^ at 5 mV/s. Figure 4b depicts galvanostatic charge–discharge (GCD) plots exhibiting similar trends, with a maximum specific capacitance of 4.5 mF/cm^2^ at 0.04 mA/cm^2^. The area under the curve related to the charge transfer kinetics increases with the rise in scan rate, as demonstrated in Fig. 4c. A higher scan rate accelerates the voltage sweep across the electrode, promoting faster electron transfer reactions and expediting the associated electrochemical processes in redox reactions. The specific capacitance was found to be 2.8 mF/cm^2^ at 100 mV/s and 6.4 mF/cm^2^ at 5 mV/s. Figure 4d shows GCD plots of LIG-Hf based devices deposited at an argon-to-oxygen ratio of 2:1 at different current densities. Higher current densities lead to quicker electron transfer during charging and discharging, reducing the overall process time. The specific capacitance for LIG-Hf-based devices was determined to be 4.5 mF/cm^2^ at 0.04 mA/cm^2^ and 3.5 mF/cm^2^ at 0.3 mA/cm^2^ with a deposition ratio of argon to oxygen set at 2:1. The specific capacitance exhibits a decrease with an increase in scan rate from 5 to 100 mV/s, as illustrated in Fig. 4e. This phenomenon is primarily attributed to the significant contribution of rapid charging and discharging of the electrochemical double layer. The specific capacitance plots obtained from galvanostatic charge–discharge (GCD) are presented in Fig. 4f, showcasing an increase in current density. The micro-supercapacitor devices based on LIG-HfO_2_ demonstrate enhanced performance at an argon-to-oxygen deposition ratio of 2:1.

The coulombic efficiency of the finalized device was calculated using Eq. (5) from the GCD graphs shown in Fig. 4d. The LIG-HfO_2_ based MSC devices have shown coulombic efficiency of 80% at 0.35 mA/cm^2^, which is in agreement with earlier published work related to MSC-based devices but lesser than bulk supercapacitors as shown in Fig. 4g^43,44^. The coulombic efficiency was found to be lesser at lower current densities and more at higher current densities in Fig. 4g may be due to the suppression of irreversible reactions^45,46^. This may be due to reactants not being able to reach the electrode surface fast enough to participate in the irreversible reaction. The highly porous structure of the LIG surface with high porosity results in low interfacial resistance and high capacity hence has improved coulombic efficiency.

### Stability, self-discharge, and impedance study of LIG-HfO_2_ devices

**Figure 5:**
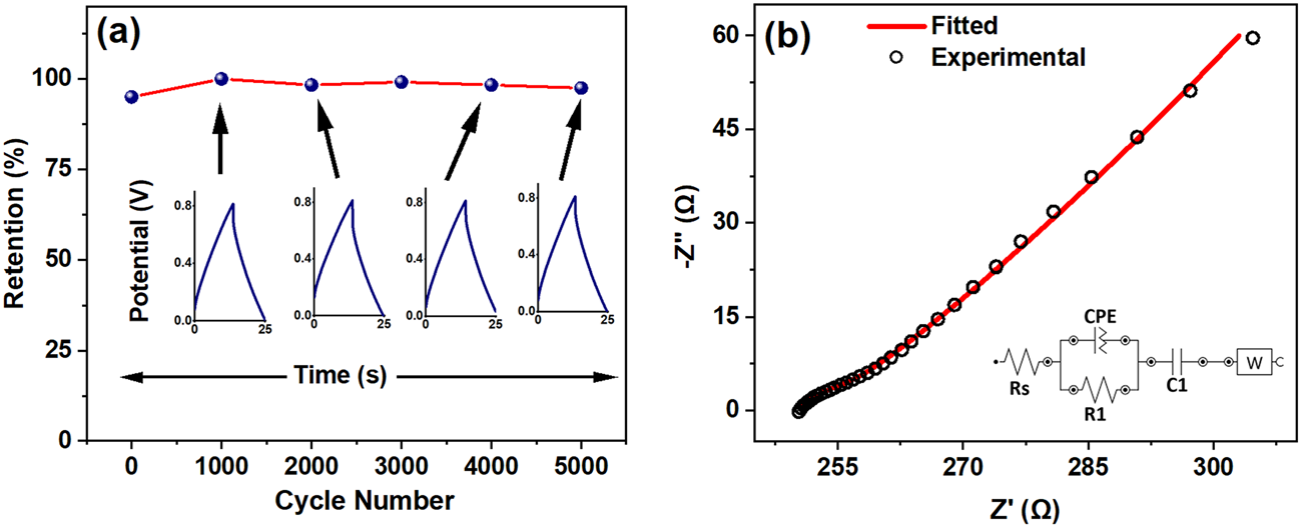
(**a**) Stability study of LIG-HfO_2_ for 5000 cycles at a current density of 0.3 mA/cm^2^, (**b**) Impedance study of LIG-HfO_2_ along with circuit fitting in the inset.

The benchmark for supercapacitor devices often revolves around the stability exhibited throughout the cycle time. Figure 5a presents the performance of LIG-HfO_2_ based devices, showcasing stability over 5000 cycles. The investigation employed galvanostatic charge–discharge voltammetry (GCD) at a current density of 0.3 mA/cm^2^ within a potential window of 0 to 0.8 V. Since charge–discharge rates tend to be slower at higher current densities, a device that proves stable at one density is generally reliable at lower densities as well. This motivated the selection of a current density covering a broader range of the device capacity to assess its real-time potential. Remarkably, LIG-HfO_2_ exhibits outstanding stability, retaining 97% of its performance after 5000 cycles. Notably, it was observed that the energy density of devices based on LIG-HfO_2_ is significantly higher. Formulas to calculate specific energy density and specific power density were utilized for a comprehensive evaluation of LIG-HfO_2_ based electrodes.

The self-discharge of LIG-HfO_2_ devices was shown in Fig. S2 (Supporting Information). The self-discharge depends on three factors 1) Ohmic leakage, 2) parasitic faradiac reaction, and 3) charge redistribution^47^. Among them ohomic leakage is the least discussed as this will also hamper the supercapacitor properties. The CV curve obtained from an ohmic leakage device will be more of a straight line passing from the origin. The main contributing terms are the parasitic faradiac reaction and charge redistribution. As discussed in the earlier section due to the presence of Hf on the surface of LIG there may be a redox reaction taking place which may also lead to a faradiac reaction. The interdigitated electrode has finger like structures along the length of the electrodes constituting the whole electrode surface. The charge stored near the beginning of fringes can be more than the later part for these interdigitated MSC devices. Such uneven charging may result in charge redistribution along the length of the electrodes contributing to reduced self-discharge time. The discharge time was more than 75% (10 min) and 52% (40 min) as shown in Fig. S2 (supporting information) which was superior to earlier published results; 70% and 38% (high voltage microsupercapacitor- HVMSCs)^48^; 65% and 37.2% (laser induced graphene-LIG based devices)^49^.

The energy density and power density were calculated following Eqs. (3) and (4) mentioned in section "Electrochemical methods". The energy density and power density were determined to be 1.45 mWh/cm^2^ and 120 mW/cm^2^, as well as 1.09 mWh/cm^2^ and 420 mW/cm^2^, respectively, at current densities of 0.1 and 0.35 mA/cm^2^. Figure 5b illustrates the impedance fitting of LIG-HfO_2_, employing a simple charge transfer model known as the Constant Phase Element (CPE) model with a mass transfer component. The comparison of the specific capacitance of LIG-HfO_2_ with other works is shown in Table 1.

**Table 1:** Comparison of specific capacitance from CV and GCD for LIG-HfO_2_ with other works.

Electrodes and materials	C_A/CV_ [mF cm^−2^]	C_A/GCD_ [mF cm^−2^]	References
MOF-LIG	8.1@1 mV s^−1^ 6.2@10 mV s^−1^	5.0@0.2 mA cm^−2^	^42^
LIG prepared from GO films	2.3@10 mV s^−1^	1.6@0.2 mA cm^−2^	^50^
LIG prepared from PI films	4.1@1 mV s^−1^	3.9@0.2 mA cm^−2^	^10^
LIG prepared from lignin films	15.4@10 mV s^−1^	12@0.2 mA cm^−2^	^51^
Laser-induced graphitization of SPEEk film	5.6@3 mV s^−1^	1.9@0.2 mA cm^−2^	^52^
Ruthenium oxide-based LIG	16@20 mV s^−1^		^53^
Ag/Ni_x_Fe_y_O_z_@rGO	–	0.22@3 µA/cm^2^/0.18@120 µA/cm^2^	^54^
Sputtered FeWO_4_	3.5@10 mV/s/2.6@100 mV/s	–	^55^
LIG-HfO_2_	6.4@5 mV/s/2.8@100 mV/s	4.5@0.04 mA/cm^2^/3.5@0.3 mA/cm^2^	This Work

### DFT calculations of LIG-HfO_2_

**Figure 6:**
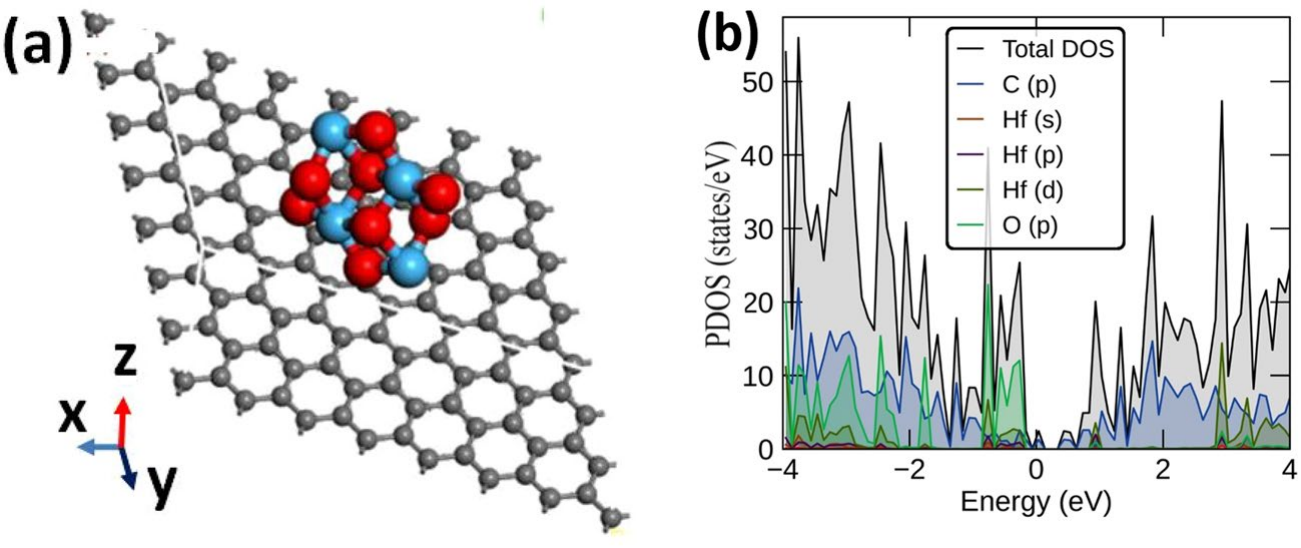
(**a**) Unit cell used for VASP calculation for LIG-HfO_2_ system, (**b**) Density of states calculation from ab inito calculations VASP for graphene and HfO_2_ based system.

The density of states for HfO_2_ deposited on LIG was calculated using VASP, as detailed in section "Computational details". In Fig. 6a, the DFT-predicted structure is illustrated, revealing a distance of 3 Å between the monolayer graphene sheet and the nearest Hf atom. The corresponding partial density of states (PDOS) is also depicted in Fig. 6b. Analysis of the PDOS spectra indicates that the Fermi level penetrates the valence band, classifying this system as a degenerate semiconductor. Notably, the primary contribution stems from the O_p_ states immediately below the Fermi level, while in the conduction band regime, C_p_ states near the Fermi level dominate. Below 2 eV in the valence band, a discernible p-d hybridization between O_p_ states and Hf_d_ states is observed. Modified graphene exhibits enhanced supercapacitor performance, attributed to heightened EDLC performance, aligning with the increased total density of states in the LIG-HfO_2_ system, as illustrated in Fig. 6b.^56^.

## Conclusion

The fabrication process for LIG-HfO_2_ based devices involved laser irradiation of a polyimide sheet, resulting in the creation of a 3D porous structure. Subsequently, the electrodes underwent further development utilizing a sputtering system with argon, with careful optimization of deposition time to ensure uniformity and enhance electrochemical performance. A deposition time of up to 1 h achieved a uniform coating on LIG-HfO_2_ fibers, as confirmed by FESEM and EDS spectroscopy, revealing a 16.6% presence of HfO_2_. However, extended deposition (1.5 h) led to agglomeration, negatively impacting supercapacitor performance, underscoring the delicate balance required for optimal LIG-HfO_2_ electrode characteristics. Detailed analysis of core lines unveiled the presence of Hf–O and Hf–C bonds. The observed increase in Hf–O intensity, the merging of doublet peaks, and the decrease in Hf-C intensity with higher oxygen exposure during deposition indicated excess oxidation of Hafnium, suggesting potential for high supercapacitor performance in LIG-HfO_2_ based devices. Indeed, LIG-HfO_2_ electrodes exhibited quasi-rectangular CV curves, indicative of favorable capacitive performance. The overall supercapacitor performance demonstrated improvement with increasing deposition time up to 1 h. Beyond this point, further deposition resulted in diminishing returns, likely attributed to increased electron transfer resistance. Variations in oxygen gas flow rates at different argon-to-oxygen ratios (4:1, 2:1, 1:1) influenced the CV plots, with the maximum specific capacitance observed at a ratio of 2:1 ranging from 2.8 mF/cm^2^ at 100 mV/s to 6.4 mF/cm^2^ at 5 mV/s. Micro-supercapacitor devices based on LIG-HfO_2_ showcased superior performance, particularly at an argon-to-oxygen deposition ratio of 2:1. The FE-SEM images demonstrated excellent stability of LIG-HfO_2_ devices up to 5000 cycles, attributing this stability to the uniform coating on the LIG fiber. The outstanding performance of LIG-HfO_2_-based MSC devices opens avenues for future exploration, suggesting the potential for other materials to be sputtering deposited on LIG to further enhance overall performance.

### Supplementary Information

Supplementary Figures.

## Data Availability

All data generated or analyzed during this study are included in this published article [and its supplementary information files].
