# Supplementary material for: Sputtered thin film deposited laser induced graphene based novel micro-supercapacitor device for energy storage application
Source: Sci Rep. 2024 Jul 15;14:16289. doi: 10.1038/s41598-024-62192-y (PMC11251010; doi:10.1038/s41598-024-62192-y)
Supplement: Supplementary file 1 — Supplementary Figures. [file 41598_2024_62192_MOESM1_ESM.docx]

**Sputtered Thin Film Deposited Laser Induced Graphene based Novel Micro-Supercapacitor Device for Energy Storage Application**

Sourav Sain^1^, Suman Chowdhury^2^, Sayantan Maity^3^, Gurupada Maity^1^, Susanta Sinha Roy^1^

^1^*Department of Physics, School of Natural Sciences, Shiv Nadar Institution of Eminence (SNIoE), Deemed to be University, Delhi-NCR, Greater Noida, 201314, India*

*^2^Department of Physics and Astrophysics, University of Delhi, Delhi 110007, India*

^3^*Department of Chemistry, School of Natural Sciences, Shiv Nadar Institution of Eminence (SNIoE), Deemed to be University, Delhi-NCR, Greater Noida, 201314, India*

Email: susanta.roy@snu.edu.in

**Supporting Information**

| 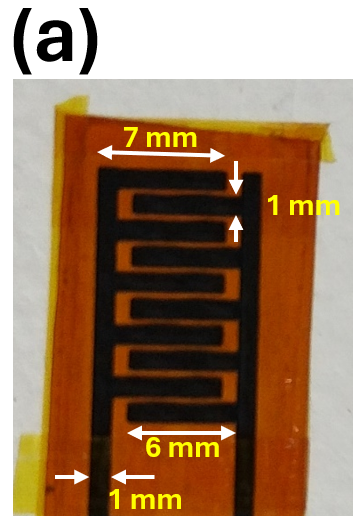 |
| --- |
| **Figure S1.** The actual image of the fabricated LIG-HfO_2_ MSC devices. |

When measuring voltage, the unavoidable charge consumption and leakage current resulting from the closed circuit were taken into account. Periodic testing was used to examine the self-discharge performance of 5.5mm-HVMSCs by testing at intervals rather than continuously sampling, which might minimize the charge loss caused by the measurement. The discharge time was obtained following earlier publications. The self-discharge voltage retention was over 75% after 10 minutes, as illustrated in Figure S2 (a).

| 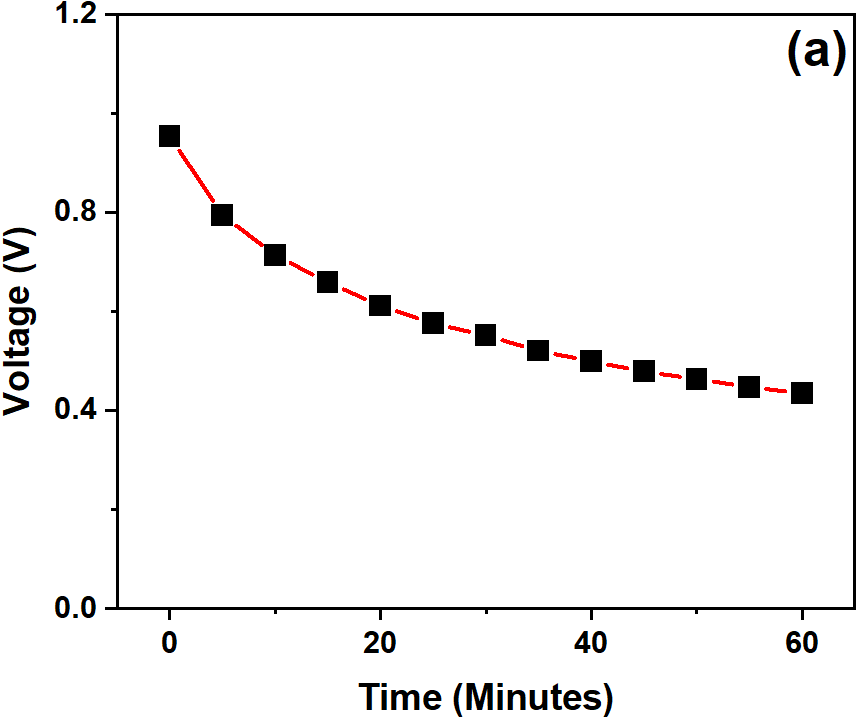 | 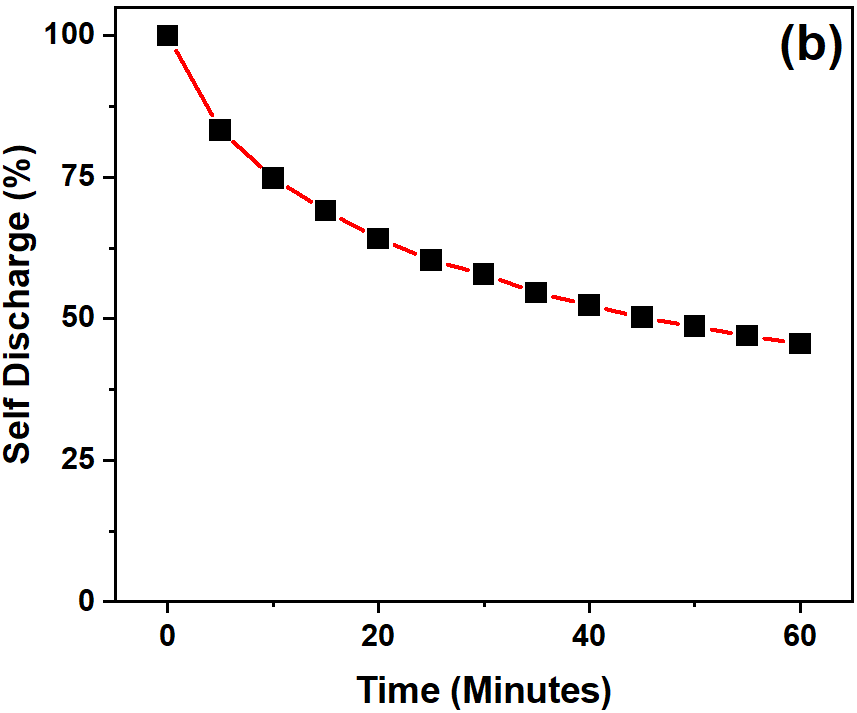 |
| --- | --- |
| **Figure S2.** The self-discharge of LIG-HfO_2_ MSC devices shows (a) Voltage with time, and (b) Rate of Voltage decrease from the initial value with time. | |
